# Decentralized, Community-Based Treatment for Drug-Resistant Tuberculosis: Bangladesh Program Experience

**DOI:** 10.9745/GHSP-D-17-00345

**Published:** 2018-10-03

**Authors:** Paul Daru, Refiloe Matji, Hala Jassim AlMossawi, Krishnapada Chakraborty, Neeraj Kak

**Affiliations:** a University Research Co., LLC, Dhaka, Bangladesh.; b University Research Co., LLC, Chevy Chase, MD, USA.

## Abstract

**Background:**

Bangladesh is a highly populous country where the prevalence of drug-resistant tuberculosis (DR-TB) is growing. With the rapid increase in DR-TB notifications through GeneXpert technology, it was imperative to come up with a new treatment strategy that could keep up with the increase of patients diagnosed.

**Intervention:**

Intervention was designed to support national transition of DR-TB management of World Health Organization-approved long course (20-to-24-month regimen) treatment from a hospital-based approach to the decentralized model of community-based programmatic management of DR-TB (cPMDT). In close coordination with the Ministry of Health and Family Welfare and National TB Program, patients were initiated into treatment at hospitals and then transferred to community-based care. A cadre of directly observed therapy providers supported treatment at the household level, supervised by the outpatient DR-TB teams.

**Methods:**

We conducted a descriptive pre- and post-intervention study of all 1,946 DR-TB patients enrolled in treatment nationwide between May 2012 and June 2015. Data were collected from hospitals, patient cards, district records, and diagnostic laboratories through the National TB Program. Intervention results were assessed in comparison with the baseline (2011) indicators.

**Results:**

During the intervention period, treatment enrollment of 1,946 diagnosed DR-TB patients through the national program increased from 50% in 2011 to 100% in 2015. The delay between diagnosis and treatment initiation decreased from 69 days in 2011 to 6 days in 2014. Most (95%) of the patients completed all scheduled follow-up smear and culture tests. By the sixth month of treatment, 99% of patients had negative smear conversion and 98% had negative culture conversion. The treatment success rate increased from 70% in 2011 to 76% in 2015 at the end of the intervention period. The results also indicate a decline between baseline and endline from 14% to 9% for patients died, 14% to 10% for loss to follow-up, and 2% to 0% for treatment failure.

**Conclusions:**

Community-based management is an effective approach for increasing access to quality-assured DR-TB treatment. Using existing structures and resources, the intervention demonstrated that favorable treatment outcomes can be achieved and sustained by treating patients with DR-TB at their homes.

## Abstract (precis)

Shifting from hospital- to community-based management of drug-resistant TB, increased treatment enrollment, reduced treatment initiation delays, improved follow-up and adherence, and lowered treatment failure, and was associated with higher cure rates and lower mortality.

## BACKGROUND

Treatment for drug-resistant tuberculosis (DR-TB)—which includes both rifampicin-resistant TB (RR-TB) and isoniazid and rifampicin-resistant TB (MDR-TB)—is currently available to only 22% of the estimated cases globally.^1^ While progress has been made to increase the number of DR-TB patients identified and reported, treatment programs continue to be hampered by waitlists and poor outcomes. The shift to decentralized, community-based care for DR-TB patients has been recommended as a means to increase the number of patients who access treatment by freeing up personnel and infrastructure at treatment-initiating sites.^2^ The effectiveness of ambulatory or community-based treatment for DR-TB has been demonstrated in a variety of settings and, under most conditions, to produce better outcomes than centralized hospital-based care.^3^^–^^6^ Decentralized treatment has a high level of acceptability among patients,^7^ contributes to a reduction in the number of patients lost to follow-up,^8^ and is less expensive than inpatient treatment.^5^ Leveraging resources and properly using existing health system structures can ensure a scalable and sustainable model for lasting impact.

Bangladesh is a densely populated country with a high burden of TB. Although the country has a relatively low prevalence of DR-TB, estimated at 1.6% among new TB cases and 29% among retreatment patients,^9^ the epidemic continues to grow, especially in younger urban populations.^10^ Although the National TB Program (NTP) started a pilot DR-TB treatment program in 2008, which was scaled up nationally by 2010, widespread availability of the treatment has lagged. The limited number of DR-TB beds in hospitals has caused long delays in treatment initiation of diagnosed patients, increasing the risk of infection and adverse treatment outcomes. With the rapid increase in the detection of drug-resistant TB by GeneXpert technology, it was imperative to come up with a new strategy that could reduce the need for hospital beds and provide diagnosed patients with quicker access to treatment.

## THE INTERVENTION

Principal barriers to the management of DR-TB cases in the pre-intervention stage included centralized treatment initiation with prolonged hospital stays up to 8 months, limited number of hospital beds for DR-TB patients, poor patient monitoring, and a lack of psychosocial support. In 2012, with support from the United States Agency for International Development (USAID) TB CARE II project, the Ministry of Health (MOH) of Bangladesh and NTP launched the community-based programmatic management of DR-TB (cPMDT) initiative to ensure access to DR-TB treatment for the increasing number of rifampicin-resistant patients confirmed with GeneXpert MTB/RIF assay testing (Cepheid, Sunnyvale, CA, USA). The cPMDT model implemented in Bangladesh was based on outpatient care of DR-TB cases after an initial and relatively short hospital treatment. The model employs patient-centered approaches to comprehensively address the various needs of DR-TB patients and improve DR-TB treatment outcomes. The key mechanisms of the model include home-based directly observed therapy (DOT), a community-based sputum collection and transportation mechanism, and a package of psychosocial support.

The framework for cPMDT was introduced to support the transition and management of DR-TB treatment from the hospital-based approach to community-based care. The framework outlined policy changes and redefined diagnostic and treatment guidelines, based on a reduced duration of inpatient treatment from the previous 6 to 8 months to less than 2 months. Through a participatory process, a standard operating procedure (SOP) for cPMDT was developed, incorporating lessons from programs in other countries and reflecting national and global guidelines.^1^^,^^11^ The SOP endorsed by the MOH and NTP provided step-by-step guidance on how to organize, implement, and monitor community-based care for DR-TB, including program planning, monitoring, and supervision. MOH and NTP, with TB CARE II team support, developed a detailed implementation plan that defined roles for DR-TB teams, outlined training curricula, and identified financial sources to roll out the program.

The cPMDT framework was introduced to support the transition and management of DR-TB treatment from hospital- to community-based care.

The project provided financial allowances to DOT providers to cover transportation costs for ensuring delivery of daily DOT and nutritional supplements to patients to promote treatment adherence. The intervention covered 38 districts and 4 city corporations. The remaining districts of the country were covered by Damien Foundation for implementation of a short-course (9 to 12 months) regimen for treatment of DR-TB cases. The cPMDT intervention supported GeneXpert testing nationwide while all diagnosed patients in Damien Foundation districts were enrolled in to the short-course treatment.

### Moving Treatment to the Community Level

The major change within the cPMDT approach was the decentralization of DR-TB service delivery from national-level hospitals to upazila-level (sub-district-level) health facilities to improve access to DR-TB services. The outpatient DR-TB teams formed at each upazila assume a central role in management of DR-TB patients at the community level. These teams are headed by the upazila health and family planning officer and consist of several members, including medical officers, the TB and leprosy control assistant, and support staff. In the last 3 years, NTP, with the project support, has formed upazila outpatient DR-TB teams in all the upazilas under the 38 cPMDT districts and has completed training of 2,340 team members on the programmatic management of DR-TB.

The upazila outpatient DR-TB team is responsible for the selection and training of DR-TB DOT providers who are officially designated to provide daily DOT and manage DR-TB patients. DOT providers are selected from the existing pool of public health workers. In areas where no public health workers are available, the program recruits and trains pharmacists and community workers from NGOs. A total of 590 health workers were trained as DOT providers during the project period.

Upazila outpatient DR-TB teams trained selected health workers to provide daily DOT and manage DR-TB patients.

Since DR-TB management capacity has been developed at the community level, MOH and NTP introduced new hospitalization and discharge criteria that recommend that DR-TB patients be transferred to cPMDT if they tolerate prescribed treatment well and sputum smear conversion is confirmed based on weekly testing. By applying these criteria, hospitals are able to discharge a majority of DR-TB patients after 4 to 8 weeks of treatment. Before discharging the patient, the treatment-initiating hospital begins the process of transferring care by notifying the respective upazila outpatient DR-TB team. The outpatient DR-TB team then identifies and trains a DR-TB DOT provider who is committed to supporting the patient and is considered acceptable to the patient. A patient transferred to community-based care now receives their daily dose from the DOT provider in their household. The outpatient DR-TB teams provide clinical support to the patient, supervise the DOT provider, and make monthly visits to monitor patient compliance with the treatment. At the subdistrict and district levels, the teams conduct monthly patient monitoring visits and provide clinical support to the patient as needed.

### Sputum Collection and Transportation

Patients transferred to community-based services need regular access to sputum testing for monitoring their response to the treatment regimen. Within cPMDT program, sputum collection points were set up at upazila public laboratories offering microscopy services. Sputum samples are sent to a reference lab for culture on a monthly or quarterly basis, or as needed. The system has improved patient compliance with follow-up sputum testing and has eliminated the need for patients to travel to reference lab. The system also supports transportation of sputum collected from presumptive DR-TB cases for GeneXpert testing at reference labs.

### Provision of Home-Based DOT

Providing patient-centered care at community level is a key element of the cPMDT model. During the treatment period, DOT providers visit each patient daily to administer injections and supervise intake of medicine according to the patient's tailored regimen. To ensure quality of care, each DOT provider is responsible for no more than 2 patients. Each DOT session is used as an opportunity for contact screening among household members and counseling on adherence, adverse events, infection prevention and control, and social support. DOT providers also monitor side effects and refer patients to upazila health complex (UHC) for any additional clinical support. Monthly clinical assessments are scheduled at the local UHC, and patients receive reminders about their routine visit schedule from their DOT providers.

### Psychosocial Support

TB patients who come from socioeconomically vulnerable groups are at increased risk of defaulting treatment.^12^ Research has shown that psychosocial and nutritional support are crucial for supporting and improving patient compliance with treatment.^13^ At the largest national chest disease hospital where most of the patients were initiated to treatment, psychosocial support to DR-TB patients, an essential component of the cPMDT model, focuses on counseling, nutritional support to patients, and vocational training. The aim of these activities is to boost patient morale to adhere to and complete the treatment. At the hospital, counseling sessions are planned for every DR-TB patient and conducted in a group setting—individual sessions are also available for patients who need additional support. The patient counseling at the community level is conducted by DOT providers during home visits. Family members of the patient are also counseled on how they could extend ongoing psychological support to the patient. As per national guidelines, each patient enrolled in community-based treatment receives a monthly stipend to promote adequate nutrition and to help cover the transportation costs of their monthly follow-up visits to the hospital. Cash transfers to patients are done through mobile banking services, which eliminates the risk of corruption and malpractice. Hospitalized patients have the opportunity to receive vocational training on tailoring, which they could use to create income opportunities. Mostly women benefited from this training.

The psychosocial support component of the cPMDT model focuses on counseling, nutritional support, and vocational training.

### Monitoring and Supervision

Upazila outpatient DR-TB teams manage the monitoring and supervision of DOT providers and DR-TB patients. They conduct monthly reviews of the performance of the DOT providers, which includes checking the patient card to verify home visits, administration of daily DOT, completeness of patient information recorded, and availability of drugs. They also make monthly visits to patient homes to monitor treatment compliance, assess patient management needs, and take follow-up actions. The upazila teams are, in turn, supported by teams at the district and divisional levels.

### mHealth Monitoring

In 2013, an mHealth application was introduced that provides a platform for the real-time monitoring of home visits and administration of daily doses by the DOT providers. Since then, each DOT provider has been equipped with a smartphone to access the Android-based application, which includes a personalized patient list and guides the provider to record key actions during each visit. Each record includes a time-and-location stamp to promote accountability, which enables supervisors to conduct online monitoring of the DOT status of every patient daily so that immediate action can be taken if any DOT was missed.

DOT providers use smartphones to access specific patient records and record key actions during patient visits.

## METHODS

Between May 2012 and June 2015, we conducted a descriptive pre- and post-intervention analysis of 1,946 DR-TB patients enrolled in 20-to-24-month treatment regimen in decentralized DR-TB care in Bangladesh. Aggregated data were collected from the NTP management information systems, which includes data from diagnostic laboratories, treatment-initiating hospitals, patient cards, and district DR-TB records to follow patients along the cascade of care and to measure the time between diagnosis and treatment initiation. Sputum and culture conversion data were routinely collected to monitor treatment adherence, efficacy, and progress. Data pertaining to training of service providers, patient enrollment in to cPMDT, and patient's compliance with DOT were retrieved from the TB CARE II project monitoring records and reports. Intervention results were assessed in comparison with the baseline (2011) indicators.

Aggregated data on treatment enrollment and outcome of DR-TB patients were downloaded into a Microsoft Excel spreadsheet. Simple quantitative analyses were performed to calculate year-wise diagnosis of patients by GeneXpert, treatment enrollment, and outcome including sputum conversion for baseline and intervention period. TB CARE II project data, maintained on a Microsoft Excel spreadsheet, were used to calculate the number of patients transferred to cPMDT for treatment and median number of days for treatment initiation delays. Treatment outcome data were analyzed for patients who completed DR-TB treatment during the scale-up of community-based care to examine the proportions of patients who were cured or completed treatment, lost to follow-up, died, or had treatment failure.

Data quality and completeness were strictly maintained by regular monitoring and supervision visits by the project field staff. Data were also regularly checked during joint monitoring visits by teams comprising representatives from NTP, the World Health Organization (WHO), and project staff. Moreover, the quality and completeness of the data were ensured by conducting regular data quality assurance assessments. No major data collection and management issues were observed except for delays updating culture results and treatment outcomes. All data collection and analysis were conducted according to international principles of maintaining privacy and confidentiality of personal information.

## RESULTS

**FIGURE 1:**
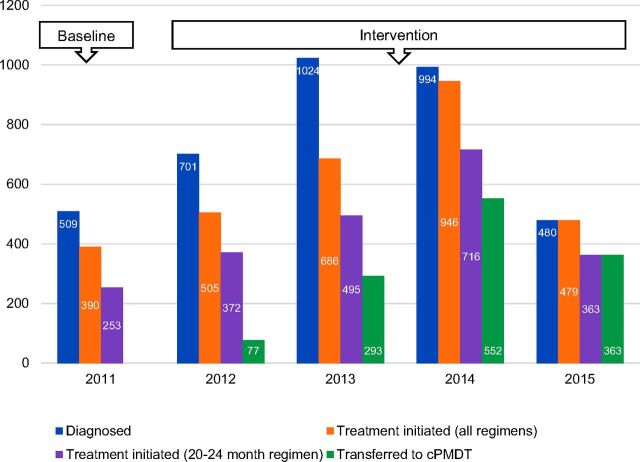
Trends in Diagnosis and Treatment Initiation, Bangladesh, 2011–2015 Abbreviation: cPMDT, community-based programmatic management of drug-resistant tuberculosis.

The transfer of DR-TB patients enrolled in 20-to-24-month treatment regimen into community care began in May 2012, and steadily increased with the geographic expansion of the cPMDT approach. The percentage of patients transferred to cPMDT increased from 21% in 2012 to 100% in 2015. This increase aligns with the annual increases in the number of DR-TB cases notified and initiated into treatment nationally. Overall, treatment initiation of patients into both long and short courses diagnosed with DR-TB increased from 77% in 2011 before cPMDT to 100% in 2015, which coincided with the transfer of all DR-TB patients into cPMDT (Figure 1).

**FIGURE 2:**
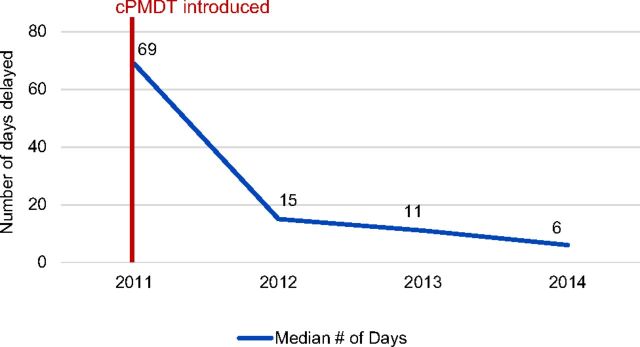
Trend in the Length of Delay Between DR-TB Diagnosis and Treatment Initiation, Bangladesh, 2011–2014 Abbreviation: cPMDT, community-based programmatic management of drug-resistant tuberculosis; DR-TB, drug-resistant tuberculosis.

Analysis of data comparing treatment initiation delay for DR-TB patients in the long course between pre- and post-cPMDT intervention showed that the median number of days lapsed between diagnosis and treatment initiation decreased from 69 days in 2011 to 15 days in 2012, 11 days in 2013, and 6 days in 2014 (Figure 2).

The delay between diagnosis and treatment initiation decreased from 69 days in 2011 to 6 days in 2014.

**FIGURE 3:**
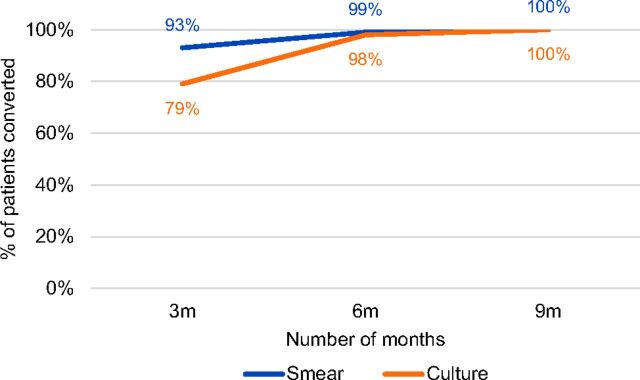
Sputum and Culture Conversion Rates of DR-TB Patients After Treatment Initiation, Bangladesh Abbreviation: DR-TB, drug-resistant tuberculosis.

Almost all (95%) of the patients completed all routine follow-up smear and culture tests, which were used to monitor patient response to treatment, specifically if and when culture conversion occurred. Of these patients, 93% were smear negative by the third month and 99% by the sixth month. Culture conversion was negative for 79% of the patients by the third month and 98% by the sixth month after treatment initiation (Figure 3).

Year-wise breakdown of data shows a gradually improving trend in treatment outcomes throughout the intervention period. Out of the 1,946 confirmed DR-TB patients enrolled into long-course treatment, 1,433 (74%) patients were successfully treated, 244 (13%) patients died, 222 (11%) patients were lost to follow-up or their data were unavailable, and 9 (0.5%) patients experienced treatment failure. From 2011 to 2015, the treatment success rate increased from 70% to 76%, while the proportions of patients who died (14% to 9%), were lost to follow-up (14% to 10%) and had treatment failure (0%) decreased (Table 1).

**TABLE.** Trend in Treatment Outcomes of DR-TB Patients During Transition to cPMDT, Bangladesh, 2011–2015

	Baseline	Intervention				
	2011 (n=240)^a^ No. (%)	2012 (n=372)^b^ No. (%)	2013 (n=495)^b^ No. (%)	2014 (n=716)^b^ No. (%)	2015 (n=363)^c^ No. (%)	Total (N=1,946) No. (%)
Cured/completed	168 (70.0)	271 (72.8)	376 (76.0)	510 (71.2)	276 (76.0)	1433 (73.6)
Died	34 (14.2)	42 (11.3)	59 (11.9)	109 (15.2)	34 (9.4)	244 (12.5)
Lost to follow-up	34 (14.2)	50 (13.4)	52 (10.5)	82 (11.5)	38 (10.5)	222 (11.4)
Failure	4 (1.7)	3 (0.8)	5 (1.0)	1 (0.1)	0 (0.0)	9 (0.5)

Abbreviations: cPMDT, community-based programmatic management of drug-resistant tuberculosis; DR-TB, drug-resistant tuberculosis.

^a^All non-cPMDT patients with 6 to 8 months hospitalization.

^b^cPMDT + non-cPMDT patients.

^c^All cPMDT patients; data from January to June 2015 only.

The treatment success rate increased from 70% in 2011 to 76% in 2015.

## DISCUSSION

The cPMDT program was a completely new approach to DR-TB patient care and management for Bangladesh. To decentralize the management and integration of service delivery with the local health care system, the initiative had to develop a national consensus on the new community-based framework for management of DR-TB and to facilitate the policy changes needed to redefine the diagnostic and treatment guidelines. Strong advocacy efforts were needed from national- and local-level stakeholders—from policy planners, program managers, and clinical experts from NTP, MOH, local Global Fund to Fight AIDS, Tuberculosis, and Malaria (Global Fund) partners, and chest disease hospitals and medical associations to community leaders and health workers—to address the concerns and ambivalence about increasing access to DR-TB services in the local context and to develop buy-in at both levels.

Before the introduction of the cPMDT program in Bangladesh, most MDR-TB patient treatment had been managed at the central hospital. The expansion of treatment to communities through the public sector provided the opportunity to significantly expand the number of DR-TB patients on treatment while maintaining high treatment quality leading to positive patient outcomes.

As the number of DR-TB cases detected continued to rise, mainly due to increased use of the GeneXpert diagnostic platform, the overall treatment initiation of DR-TB patients through both long- and short-course regimens started to rapidly increase—from 50% in 2011 to 100% in 2015. The proportion of DR-TB patients who initiated the long-course regimen, which was the intervention focus, increased from 67% in 2011 to 76% in 2015. The remaining 24% were enrolled in the short-course regimen supported by the Damien Foundation. Of the 1,946 DR-TB patients who initiated treatment between 2012 and 2015, 1,285 (66%) received and completed treatment in their communities. This change was crucial, as it eliminated the backlog of patients awaiting treatment initiation—a major concern in the pre-cPMDT stage—and the associated risk of infection at the facility and community levels.

While there are few published studies associating poor treatment outcomes with delayed treatment initiation, evidence of programmatic changes leading to shorter treatment initiation delays does exist.^14^ Prior to the introduction of cPMDT, the median wait time between diagnosis and treatment initiation was 69 days in 2011. In 2014, the median wait time decreased to 6 days. Several factors contributed to this change. The transfer of patients to their communities after about 2 months of hospitalization enhanced the capacity of treatment-initiating hospitals to rotate a single bed for up to 6 patients a year, compared with 1.5 patients in previous years. Additionally, expanding the number of treatment-initiating hospitals at regional headquarters, increasing the number of hospital beds allocated to DR-TB patients, and increasing the efficient management and coordination of processes related to treatment initiation, release, and transfer of patients to community added speed to each step, reducing delay while increasing access to treatment for more patients.

Sputum culture conversion is an important interim indicator of the efficacy of MDR-TB treatment as well as an important predictor for treatment outcome.^15^ Monthly culture monitoring is essential for early detection of treatment failure in patients with MDR-TB.^16^ The project routinely tracked culture and smear conversion of patients to identify delayed converters and provide additional treatment support to them. Data analysis showed that 95% of the patients in community-based care complied with the requirement to have smear and culture tests done monthly and, after sputum conversion, culture tests done quarterly. Within 6 months of treatment, smear and culture conversion rates for patients reached 99% and 98%, respectively. The keys to ensuring patient compliance with the follow-up test requirements, were the decentralization of treatment to the community level and real-time monitoring of patients through a web-based mHealth application.

Compared with global averages, DR-TB patients in Bangladesh experienced better treatment outcomes. The treatment outcome data, measured at the end of the project in 2015, showed a treatment success rate of 76%, which is much higher than global average of 54%.^17^ Only 10% of the Bangladesh patients were lost to follow up compared with 21% globally^17^; and 0.5% patients in Bangladesh experienced treatment failure compared with 8% globally.^17^ In 2015, all 363 DR-TB patients received and completed treatment in their respective communities. The project results suggest that community-based care of DR-TB patients can achieve high levels of treatment adherence and favorable treatment outcomes.

A systems strengthening approach with a focus on integrated service delivery was a major consideration for financial sustainability of the cPMDT intervention. The intervention was planned consciously to avoid building a parallel system that would be difficult to sustain after the end of the project. However, the system will require additional resources to support recurring costs for continued training of service providers, procurement of drugs for treatment of DR-TB, provision of financial allowances to DOT providers and patients, quality assurance, and monitoring and supervision. The MOH and NTP are cognizant of the additional resource needs and expect to secure greater allocation of revenue funds from the government to cover certain costs. The monthly stipend to DOT providers and patients, initially supported through USAID funds, has already been shifted to the Global Fund. Stronger advocacy efforts are needed to mobilize more resources through the Global Fund, international donors, and local private sector entities to effectively continue and expand the DR-TB initiative.

Increased resources are needed through government revenue funds, the Global Fund, international donors, and local private entities to effectively sustain the DR-TB initiative.

### Challenges and Lessons Learned

As a new approach for Bangladesh, the cPMDT project had to overcome several challenges, most of which were addressed through consistent effort and effective coordination with NTP, WHO, and other local-level partners. Effective planning and coordination for rolling out the program to the community level required a considerable effort to train and mobilize hundreds of skilled personnel at the upazila and community levels to manage and monitor patients integrated with the existing health care system. Establishing well-functioning outpatient DR-TB teams that would be responsive to their new roles and responsibilities for management of DR-TB patients seemed to be a big challenge. The participation and combined effort from the local health authority, DR-TB hospitals, and NTP were essential to overcome those challenges. The existing health care system was not ready to take full responsibility for sustaining the initiative without external vigilance and support. The project's facilitative role, including close monitoring of field activities, was key to ensuring that patients were transferred without delay to the community and that they remained adherent to the treatment regimen.

### Limitations

The study presented here has several limitations. Because we had to rely on the routine data collected by NTP for the national DR-TB program, which did not disaggregate cPMDT and non-cPMDT patients, we were unable to directly compare results between the two groups making it difficult to determine the impact of cPMDT model on the treatment outcome of DR-TB patients. We were also unable to compare results of sputum conversion between pre- and post-intervention because the data prior to the intervention were not available.

A concurrent health system strengthening effort may have also impacted the results of the intervention. During the cPMDT implementation, inpatient facilities were improved and the number of hospital beds for DR-TB treatment increased through a separate health system strengthening program, contributing to increasing national capacity for and access to DR-TB treatment.

## CONCLUSION

The implementation of cPMDT in Bangladesh has greatly increased the proportion of DR-TB patients enrolled in treatment, reduced the delay in treatment initiation, and improved treatment adherence and outcomes among patients. The model demonstrates that cPMDT is an effective approach for increasing access to and providing quality-assured DR-TB treatment. A high cure rate with minimal default and failure is achievable by treating patients in their homes, where they feel more comfortable and receive family support. As detection of DR-TB cases continues to increase with the use of rapid diagnostic technologies, stronger systems for decentralized patient management are needed to accommodate treatment needs. Unless hospitalization is necessary for clinical reasons, treatment of DR-TB patients in the community from the first day is the way Bangladesh can mitigate the severe clinical, social, and economic consequences of DR-TB for individuals and communities.

The cPMDT initiative has strengthened Bangladesh's health system by increasing the number of patients enrolled in treatment, reducing treatment delay, and improving patient adherence and outcomes.

## Notes

**Peer Reviewed**

**Funding:** This study was made possible by the support of the American people through the United States Agency for International Development under the TB Care II Cooperative Agreement Number AID-OAA-A-10-0002, managed by University Research Co. (URC).
