# Corrigendum: Daru et al., Decentralized, Community-Based Treatment for Drug-Resistant Tuberculosis: Bangladesh Program Experience

**DOI:** 10.9745/GHSP-D-18-00398

**Published:** 2018-12-27

See corrected article.

In the article, “Decentralized, community-based treatment for drug-resistant tuberculosis: Bangladeshprogram experience,” by Paul Daru et al. (Volume 6, Number 3), the results section of the Abstract incorrectly cited the baseline percentages of patients who died and were lost to follow-up as 34%. These baseline figures have been corrected to 14%, per the data reported in the Table and main body of the article.

